# Research on the effects of curvature and shear keys on torsional stiffness and interfacial stress of segmented U-shaped curved bridge

**DOI:** 10.1038/s41598-025-19337-4

**Published:** 2025-10-09

**Authors:** Shuai Zhang, Chuang Wang, Hongchun Qu, Wenjie Xu, Lin Xiao, Qingsong Fan, Chuanbiao Wang, Yanqun Han

**Affiliations:** 1 School of Civil Engineering, Central South University, Changsha, 410075 China; 2 China Communications Construction Company, Second Harbor Engineering Co., Ltd, Wuhan, 430040 China; 3 The Second Xiangya Hospital, Central South University, Changsha, 410011 China; 4 Chongqing Transportation Planning and Technology Development Center, Chongqing, 400074 China

**Keywords:** Segmental u-shaped curved bridges, Curvature radius, Shear keys, Torsional stiffness, Interfacial stress, Engineering, Materials science

## Abstract

This paper investigates the segmental U-shaped curved bridge of Bogotá Metro Line 1 project in Colombia. The effects of curvature radius (*R*) of the bridge and shear keys (number, arrangement, and size) at inter-segment bonding joints on the bridge’s torsional stiffness and interfacial stress are analyzed. The results demonstrate a nonlinear relationship between bridge curvature and torsional stiffness. As the curvature radius increases, torsional stiffness enhances while the growth rate declines gradually. Increasing the number of shear keys in the web and floor regions of U-shaped bridge segments, along with extending their width, effectively enhances the bridge’s torsional stiffness and global deformation resistance while ameliorating the interfacial stress state between segments. These findings offer both theoretical and practical guidance for the design of segmental U-shaped curved bridges and shear key systems.

## Introduction

In recent years, Rail transit industry has developed rapidly, and various forms of beams have been widely used in this field, such as U-shaped bridges, T-shaped beams, box beams, and so on^1,2^. Compared to other beams, U-shaped bridges have many advantages, such as better noise reduction effects due to their lower construction height, aesthetic appearance, and high economic benefits. Therefore, U-shaped bridges are widely used in the rail transit field^3–7^.

In 1962, a bridge crossing over the Seine River in France was constructed—regarded as the inaugural precast segmental concrete bridge on a global scale. Segmental bridges have garnered significant attention due to their numerous advantages, including fast construction speed, environmental friendliness, and superior quality control. By adopting this technology, construction time and traffic disruptions can be significantly reduced, thereby greatly enhancing economic benefits^8–10^. Against the backdrop of increasing global demand for bridges, the appeal of segmental bridges is growing^11,12^. For segmental bridges, joints are the weak points in their structure, thus they require special attention and handling^13^. This is because the strength, maintainability, and structural behavior of precast concrete segmental bridges largely depend on the performance of the joints between the segments. There are various existing forms of joints, including dry joints, wet joints, and epoxy resin joints. Compared to other types of joints, epoxy resin joints are considered to perform better in terms of durability and ultimate shear load capacity. The use of epoxy resin can in-crease the shear load capacity of the joint^14,15^. Additionally, uncured epoxy resin can act as a lubricant for the joint, compensating for irregularities between the joint surfaces^16^.

In bridge engineering, curved bridges are widely used to adapt to terrain and traffic requirements. However, in practical use, curved bridges face serious torsion problems, which can affect the stability and safety of the bridge, and in severe cases may lead to structural overturning and damage^17^. The case of overturning involving the Yangmingtan Bridge exemplifies a critical vulnerability inherent in bridges with curvature. Observable is that, when subjected to eccentric loading conditions, such curved structures are notably susceptible to experiencing torsional instability^18^. Therefore, significant to in-depth research are the torsional stiffness attributes inherent within curved bridges, and from enhancements of anti-overturning capabilities can overall safety appreciably benefit.

Many scholars have conducted research on the bending and shear behavior of bridges, but studies on the torsional behavior are relatively fewer in number. Meyyada et al.^19^ conducted a review on the torsional strengthening of reinforced concrete (RC) beams using externally bonded (EB) composite materials and established a database for RC beams retrofitted with EB fiber-reinforced polymer (FRP) and fiber-reinforced cementitious matrix (FRCM). Zhao et al.^20^ discussed the torsional behavior of a novel reinforced concrete U-shaped steel-concrete composite beam (RCUCB) system through a series of torsion tests. Mane et al.^21^ proposed a high-yield-strength welded wire mesh that can be wrapped around concrete members to improve composite performance and avoid sudden cracking, while also increasing the torsional stiffness of reinforced concrete beam specimens.

Numerous scholars have conducted research on the shear keys of segmental bridge joints. Jiang et al.^22^ tested full-scale specimens with shear keys under different levels of confining stress and proposed a shear failure mechanism for the continuous failure of multiple shear keys in dry joints based on the experimental results. Celia et al.^23^ designed a new push-off dry joint test under combined forces, testing 24 push-off dry joint specimens under axial force, bending force, and shear force, and concluded the effects of parameters such as the number of shear keys, confining stress level, and eccentricity on the shear resistance of segmental bridges. Hua et al.^24^ designed and developed 3D-printed concrete shear keys with epoxy mortar infill and studied the various designs of 3D-printed concrete shear keys with different shear key angles and shear joint inclinations through push-off and slant shear tests.

Compared with straight bridges, curved bridges exhibit more complex stress patterns, and the problem of eccentric loads and torsion is more prominent. At present, the research on segmental U-shaped curved bridges is not sufficient, especially the effects of the arrangement, number and size parameters of shear keys and different curvature radii (*R*) of bridge on the torsional stiffness and interfacial stress of the bridge are not systematically studied in combination with the construction characteristics of segmental assembly and the section characteristics of U-shaped bridges. The above effect mechanism can provide theoretical support for the design optimization and engineering application of segmented U-shaped curved bridge, and promote the technical development and promotion of this kind of bridge structure.

## Research background

**Fig. 1:**
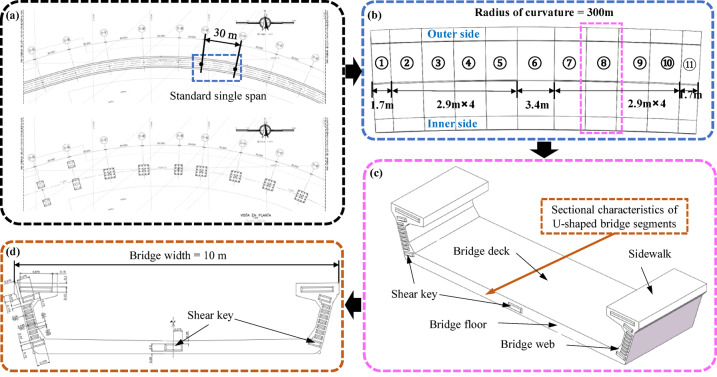
Overview of the bridge and details (unit: m): (**a**) Bridge plan view; (**b**) 30 m span U-shaped spliced beam (11 segments); (**c**) Detail drawing of a segment; (**d**) Shear key details.

This study relies on the Bogotá Metro Line 1 bridge project, which is designed with a total length of 23.86 km and a total of 742 span bridges. The segmental U-beam assembly process is adopted, of which the curved bridge has 180 spans. Overview of the bridge and details is shown in Fig. 1, the standard span of the bridge in the project is mainly displayed, which is 30 $\:\text{m}$ long and 10 $\:\text{m}$ wide, and consists of 11 segments. The *R* of the bridge in Fig. 1 is 300 m. The effects of various factors, including the arrangement, number, size of shear keys and curvature of the bridge, on the torsional stiffness and interfacial stress of segmented U-shaped curved bridges are investigated using numerical methods in this paper. When other conditions remain unchanged, a change in the deformation difference between the inner and outer sides of the bridge caused by altering a single parameter indicates a change in its torsional stiffness. Therefore, this paper characterizes torsional stiffness by the “deformation difference between the inner and outer sides”.

## Finite element analysis model

**Fig. 2:**
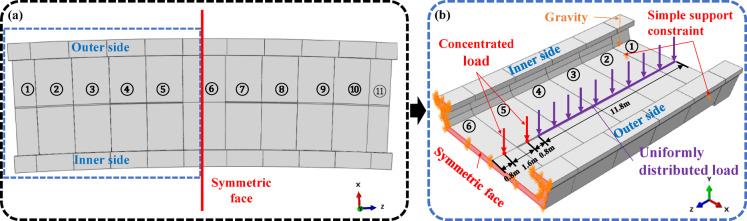
(**a**) Full-bridge model; (**b**) Half-bridge model, boundary and load.

Due to the symmetry of the structure, the equivalent simplification principle in structural mechanics is adopted to select the half-bridge (15 m) of the full-bridge (30 m) as the research object, and the half model is used to simplify the calculation process, as shown in Fig. 2(a) and (b). The finite element analysis is performed using ANSYS.

The boundary conditions and load of the half-bridge are shown in Fig. 2 (b). The symmetric boundary conditions are applied to the symmetric face, and the bearings at the end of the bridge are simply supported. The structure is subjected to uniform load of 48 k N/m, two concentrated loads of 150 k N and standard gravity. The load conditions adopted in this paper are determined with reference to the *Code for train load diagrams* and are consistent with the basic load framework of the Bogotá Metro Line 1 project^25^. Since the core of this study is to analyze the impact of structural parameters (such as curvature radius, shear key configuration, etc.) on torsional stiffness and interfacial stress, within the scope of this research, as long as the load conditions of each model are kept within a reasonable range and consistent, the effects caused by structural changes can be effectively compared.

**Fig. 3:**
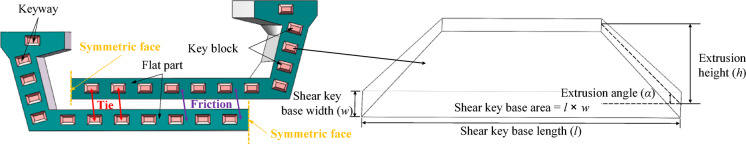
Contact mode and details of shear key parameters.

The single-span bridge with 11 segments of U-shaped bridge girder contains 10 joints in total. as shown in Fig. 1. Joints between bridge segments are bonded with epoxy resin adhesive. Tie constraint (Pink section) is used to connect the key block and keyway of the shear key, while friction contact(Green section) is used to simulate the flat part of the joint, and 0.45 is set as the friction coefficient^26^as shown in Fig. 3. The contact mode has been validated by Zhang et al.‘s research—through comparisons of extensive experimental and simulation data, they confirmed its rationality, with most groups showing an error within 10%. However, the accuracy of the contact mode is still limited, as a small number of groups exhibit an error exceeding 20%^26^. In actual engineering, the mechanical properties of the epoxy adhesive layer fluctuate with environmental temperature and humidity, while the model simplifies it to uniform contact properties, which may lead to deviations in interfacial stress calculation. Additionally, the Tie constraint assumes perfect fitting between the shear key and keyway, without considering potential installation gaps during construction, which may underestimate the relative slip between segments. Although the impact of these factors is limited, the setting of the contact mode remains the main source of error in the numerical simulation of this study, and further exploration of more accurate contact models is required in subsequent research.

The shear key of the finite element model are set to the same specification in this paper, which is more convenient to discuss the effect of the shear key on the torsional stiffness and interfacial stress of the bridge under the control of different parameters^11^. The parameters of the shear keys are marked in Fig. 3.

**Fig. 4:**
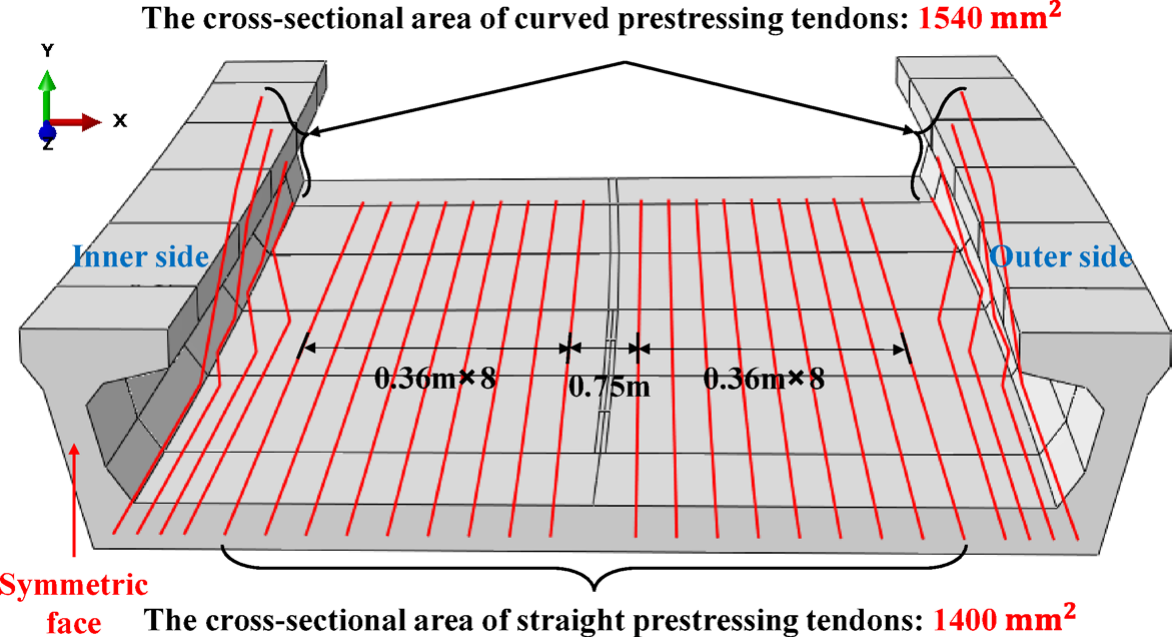
Prestressed tendons distribution.

The elastic constitutive relation of concrete and prestressed tendons is used in finite element simulation. The concrete strength class is C50. The prestress applied to the prestressed tendons is 1395 MPa, the cross-sectional area of curved prestressed tendons is 1540 mm², and that of straight prestressed tendons is 1400 mm². All data refer to the engineering drawings of the Bogotá project. Material parameters detailed in Table 1. The layout of the prestressed tendons is shown in Fig. 4.

**Table 1:** Material parameters.

Material	Elastic modulus (GPa)	Poisson’s ratio	Density(kg/m^3^)
Concrete	35.04	0.2	2500
Prestressed tendons	206.0	0.3	7850

**Fig. 5:**
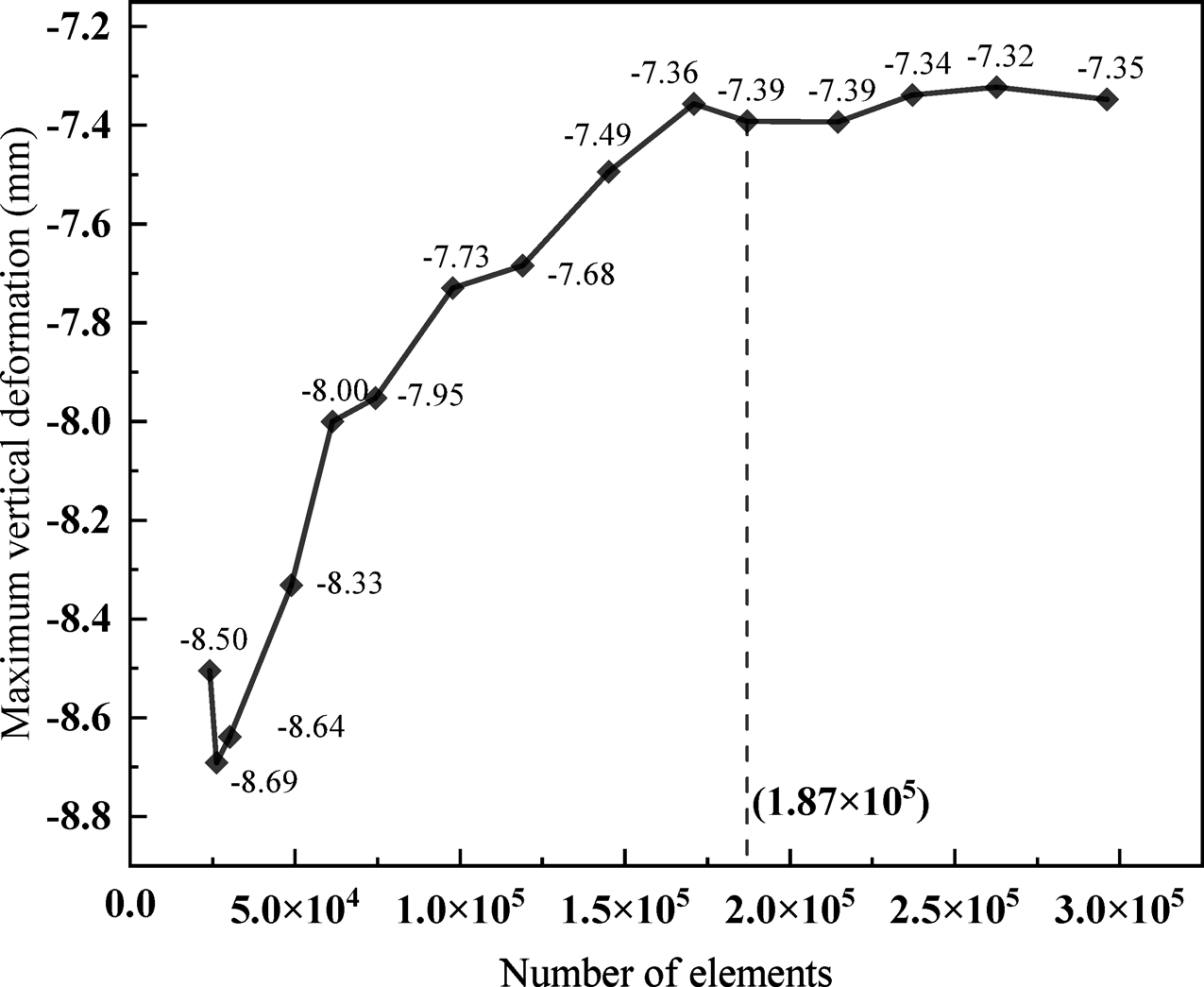
Mesh convergence analysis.

To ensure the accuracy of the finite element simulation in this study, a mesh convergence analysis is carried out based on a model with *R* = 200 m, *l* = 300 mm, *w* = 200 mm, *h* = 50 mm, *α* = 45°, and 24 shear keys. The shear keys are uniformly distributed on the web and floor of the bridge, as shown in Fig. 3. The maximum vertical deformation (deformation along the Y axis) of the bridge is calculated for multiple sets of models with varying numbers of elements. As shown in Fig. 5, the results indicate that as the number of elements in the model increases, the maximum vertical deformation of the bridge gradually stabilizes, thereby confirming the validity of the finite element simulation. Considering the calculation speed and accuracy, 1.87 × 10^5^ elements are finally selected as the number of calculation elements.

## The effect of the curvature of curved bridges on torsional stiffness and interfacial stress

To investigate the effect of bridge curvature radius on the torsional stiffness and interfacial stress, three different finite element calculation models are established by changing *R*. The parameters corresponding to models with different curvature radii are shown in Table 2, which all come from engineering drawings. The arrangement of shear keys for the models in Table 2 is shown in Fig. 3. The shear keys are uniformly distributed on the web and floor of the bridge.

**Table 2:** Parameters corresponding to models with different curvature radii.

Name	*R*(m)	Number	l (mm)	w (mm)	h (mm)	α (°)
*R =* 200	200	24	300	200	50	45
*R =* 300	300	24	300	200	50	45
*R =* 400	400	24	300	200	50	45

**Fig. 6:**
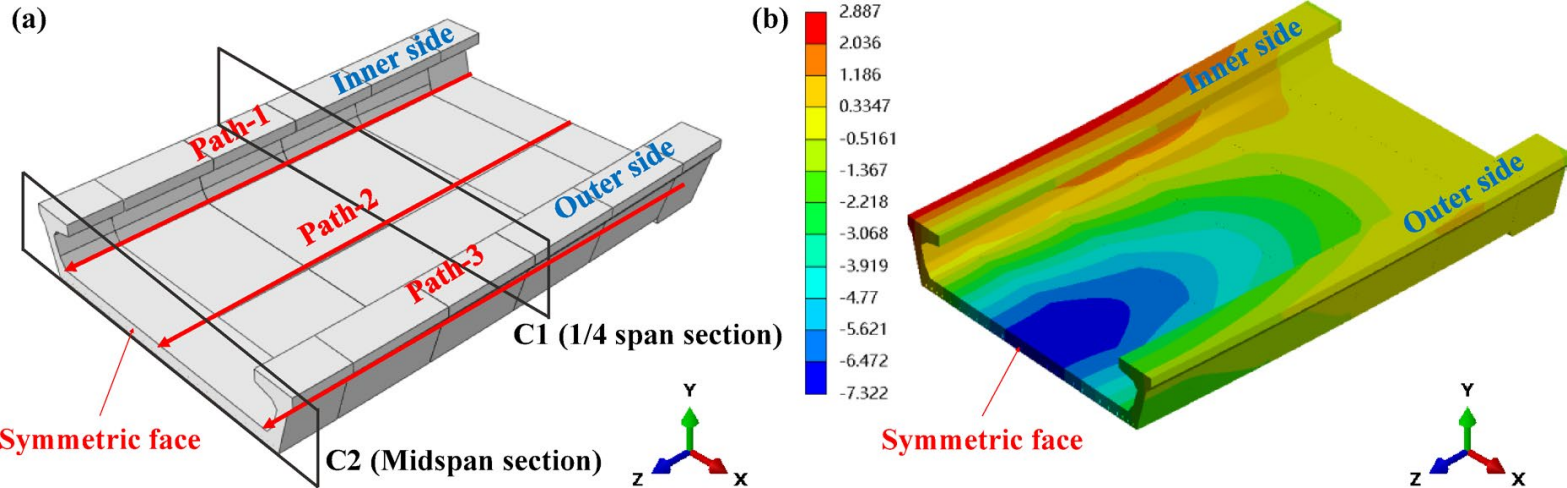
(**a**) Bridge paths and section settings; (**b**) Vertical deformation nephogram.

**Fig. 7:**
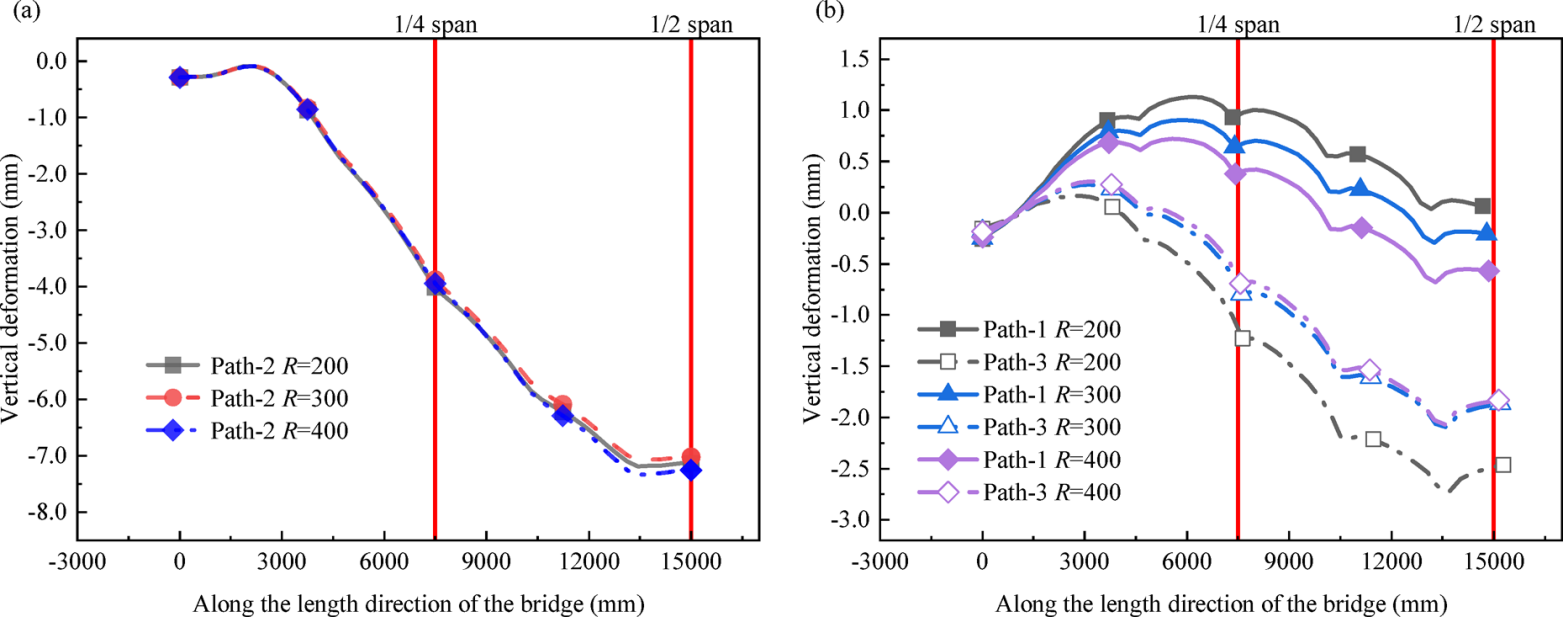
Deformation of curved bridge models with different curvature radii: (**a**) Path-1 deformation; (**b**) Path-1 and Path-3 deformation.

**Fig. 8:**
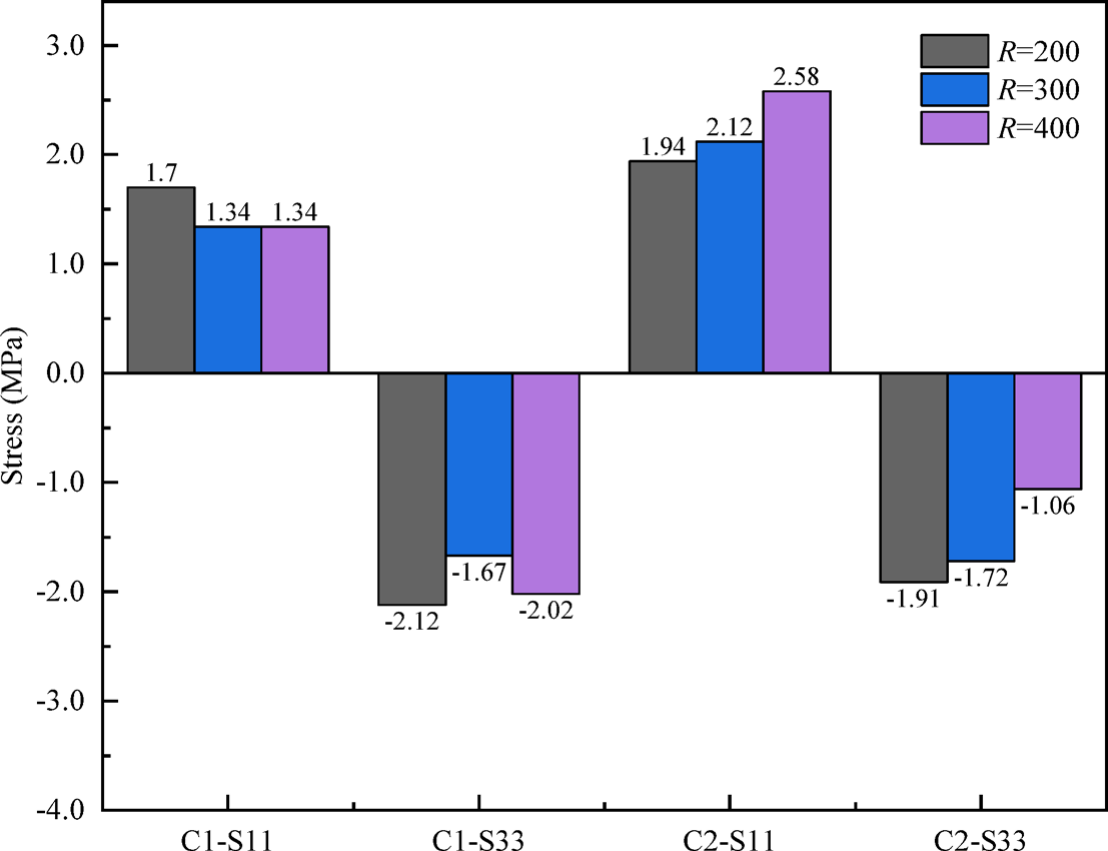
Stress Distribution in Curved Bridge Models with Different Curvature radii.

To facilitate result analysis, three paths (Path-1, Path-2, Path-3) and two cross - sections (C1 and C2) are set on the curved bridge deck, as shown in Fig. 6(a). Path-1, Path-2 and Path-3 are used to obtain the inner, central and outer deformation of the bridge from the inside to the outside. C2 is in the midspan and C1 is in the quarter span of the bridge. The results of three different curvature radii models are shown in Figs. 7 and 8.

The overall vertical deformation nephogram of *R* = 200 model is shown in Fig. 6(b), and the corresponding nephogram of other models are similar. The overall deformation of the structure is asymmetric. The bridge deforms upwards along Path-1 and deflects downwards along Path-3, indicating a clear trend of torsional deformation, which is likely due to the structural characteristics of curved bridges. The outer side of a curved bridge is longer, and its relative weight has a greater impact, with a higher stiffness on the outer side. And the inner side is short, with less impact on relative weight and relatively low stiffness. When subjected to the same prestress, the effect of the inner prestress is more pronounced, resulting in upward deformation on the inner side. On Path-2, the downward vertical deformation of the bridge from the simply supported end to the midspan gradually increases, and reaches the maximum at the midspan.

As shown in Fig. 7, under the same load conditions, the vertical deformation of different curvature radius models along Path-2 is basically the same. However, as the curvature radius increases, the vertical deformation of both the inner and outer sides of the bridge decreases significantly, and the difference in deformation between the inner and outer sides also decreases. When the curvature radius increases from 200 m to 300 m and 400 m, the difference in deformation between the inner and outer sides at the quarter span decreases by 34.04% and 50.64%, respectively, while that at the mid-span decreases by 34.38% and 49.84%. Sudden changes may occur on the curve due to the structural characteristics of different beam joints.

According to Vlasov’s thin-walled curved beam theory, the additional torque induced by curvature has a nonlinear relationship with curvature—in the small curvature range, this relationship is specifically proportional to 1/*R*^2^. When the radius of curvature is small, the inner and outer flanges of the thin-walled cross-section exhibit significantly different stress states due to the bending-torsion coupling effect: the inner flange bears greater compressive stress, while the outer flange bears larger tensile stress, leading to the offset of the cross-sectional neutral axis and extremely uneven stress distribution. This unevenness reduces the effective torsional moment of inertia of the cross-section, thereby causing a significant decrease in the torsional stiffness of the beam. When the radius of curvature exceeds a certain threshold, the bending-torsion coupling effect weakens, and the mechanical behavior of the beam gradually approaches that of a straight beam. At this point, the torsional stiffness is mainly determined by the torsional constant of the cross-section itself, and the rate of increase in stiffness with the increase of the radius of curvature slows down significantly^27,28^.

The stress conditions of C1 and C2 in curved bridges with different curvature radii are showed in Fig. 8. For C1, the change of the interfacial stress of the bridge with the curvature radius is not obvious, and the overall performance is that the interfacial stress increases with the decrease of the curvature radius. For C2, as the curvature radius increases, the average tangential stress along the x-axis (S11), which is obtained by weighted averaging of the x-axis stress components of all solid elements within the cross-section with element area as the weight, shows an upward trend, while the absolute value of the average normal stress along the z-axis (S33) gradually decreases.

## The effect of shear keys on torsional stiffness and interfacial stress

The joints between bridge segments are equipped with multiple pyramid shaped shear keys. The number, arrangement, and size of these shear keys can all affect the torsional stiffness and interfacial stress of segmental U-shaped curved bridges. The effects of different shear key configurations on the torsional stiffness and interfacial stress of segmental U-shaped curved bridges are investigated. In this study, the setting of the number, size and distribution of shear keys strictly follows the core principle of engineering research—single variable control. When studying the influence of a single parameter, other parameters are ensured to remain unchanged, so as to clearly reflect the effects brought by the change of this parameter, and the specific values of the number and size of shear keys are formulated in accordance with this rule^11,26,29^.

### The effect of shear key number

**Fig. 9:**
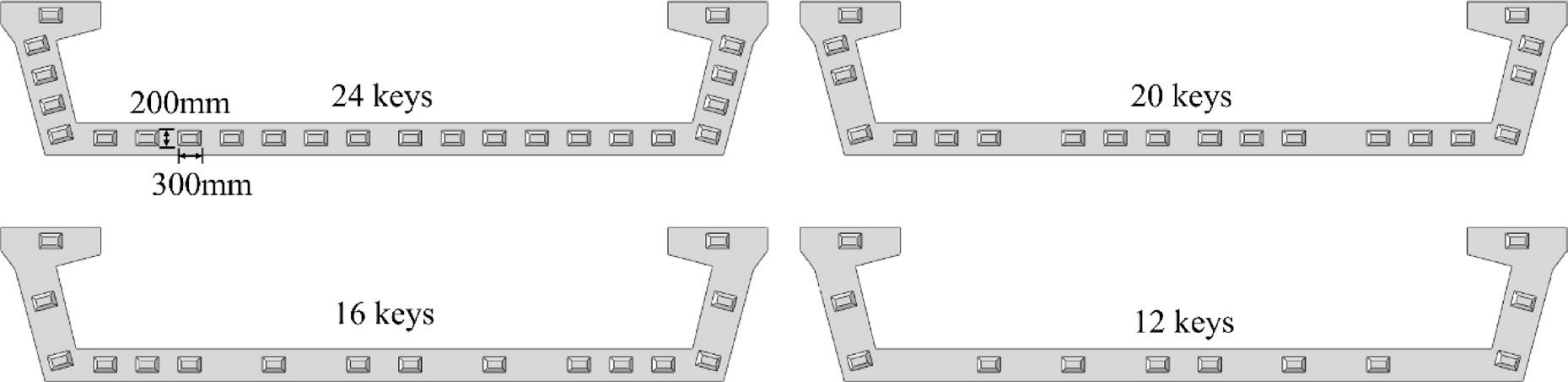
Layout of different number of shear keys.

**Fig. 10:**
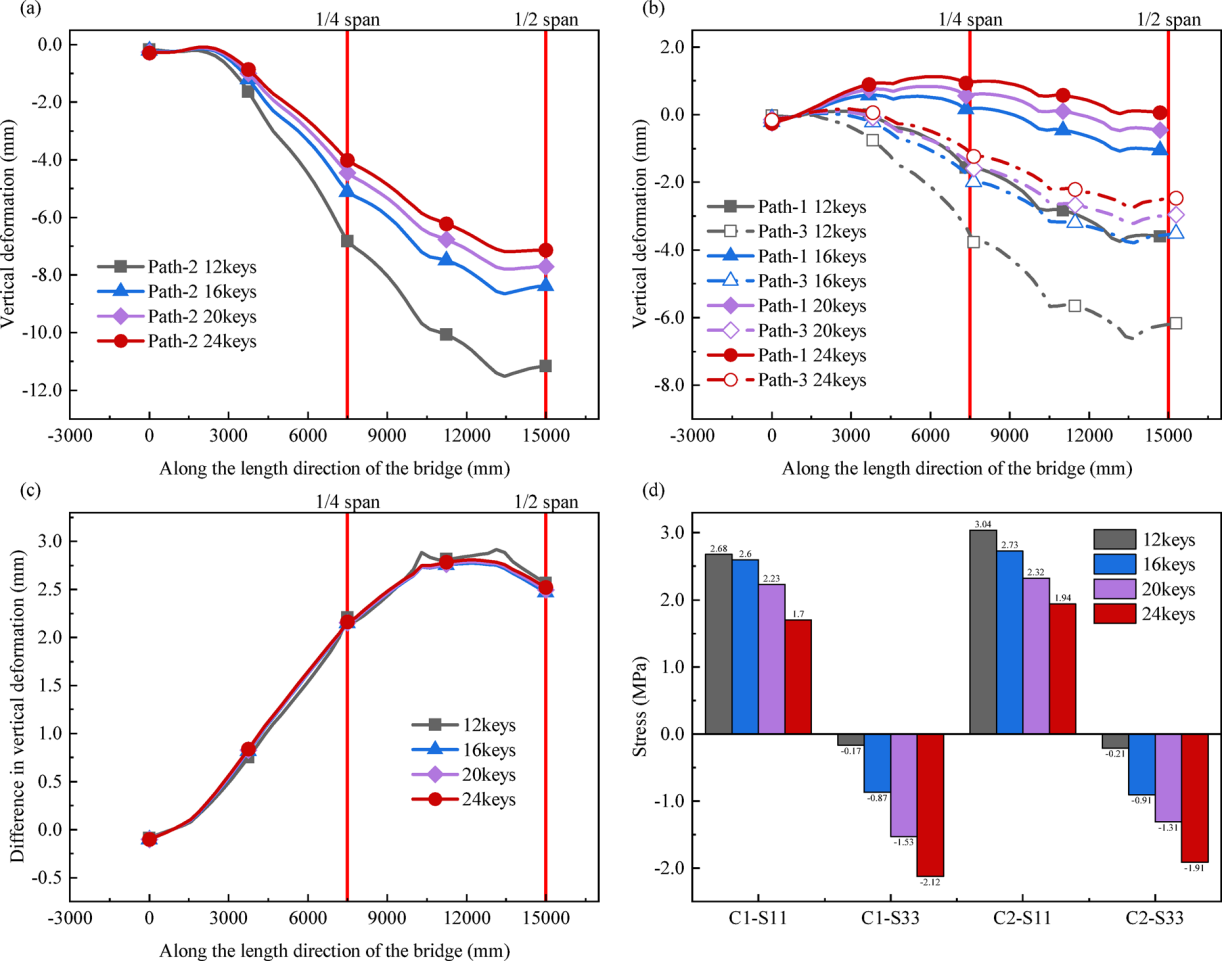
Effect of different shear key numbers on bridge model calculation results: (**a**) Vertical deformation along Path-2; (**b**) Vertical deformation along Path-1 and Path-3; (**c**) The difference in vertical deformation along Path-1 and Path-3; (**d**) Stress at different bridge interfaces.

Four different finite element calculation models are established by changing the number of shear keys. The parameters corresponding to models with different numbers of shear keys are shown in Table 3. The four types of interface shear key arrangement between bridge segments are established according to Table 3, as shown in Fig. 9. The shear keys are uniformly distributed on the web and floor of the bridge. 12keys, 16keys, 20keys and 24keys models are used for finite element simulation analysis. The results for these four models are presented in Fig. 10.

**Table 3:** Parameters corresponding to models with different number of shear keys.

Name	*R*(m)	Number	l (mm)	w (mm)	h (mm)	α (°)
12keys	200	12	300	200	50	45
16keys	200	16	300	200	50	45
20keys	200	20	300	200	50	45
24keys	200	24	300	200	50	45

As shown in Fig. 10 (a) and (b), when the number of shear keys increases from 12 to 24, the vertical deformation of the model along the three paths gradually decreases. Taking path-2 as an example, compared with key 12, the vertical deformation of keys 16, 20, and 24 at the quarter span is reduced by 25.07%, 34.73%, and 41.25%, respectively. At mid-span, the reductions are 24.93%, 31.06%, and 36.18% respectively. Results show that increasing shear key number effectively controls vertical deformation of segmental U-shaped curved bridge. Notably, the deformation reduction when the number of shear keys increases from 12 to 16 is significantly greater than the reduction during the stage from 16 to 24 keys. Considering the manufacturing difficulty and cost factors, the lowest reasonable number of shear keys should be matched for different segmented U-shaped curved bridge.

As shown in Fig. 10 (b) and (c), with the increase of shear keys, the vertical deformation on the inner or outer side decreases, but the difference in deformation between the inner and outer sides remains basically unchanged. Therefore, changing the number of shear keys will not significantly change the overall torsional stiffness of the bridge.

Figure 10 (d) shows the effect of different shear key numbers on the interfacial stress of the bridge. With the increase of shear keys, for C1 and C2, S11 decreases, and the absolute value of S33 increases and becomes negative, indicating that the interface is under overall compression along the bridge direction. Therefore, increasing the number of shear keys can enhance the overall integrity of the bridge and improve the shear bearing capacity at the joints.

Increasing the number of shear keys in the design can improve the connection stiffness of the bridge and effectively improve the interfacial stress state of the bridge structure. However, increasing the number of shear keys has little effect on improving the torsional stiffness of the bridge.

### The effect of shear key arrangement

**Fig. 11:**
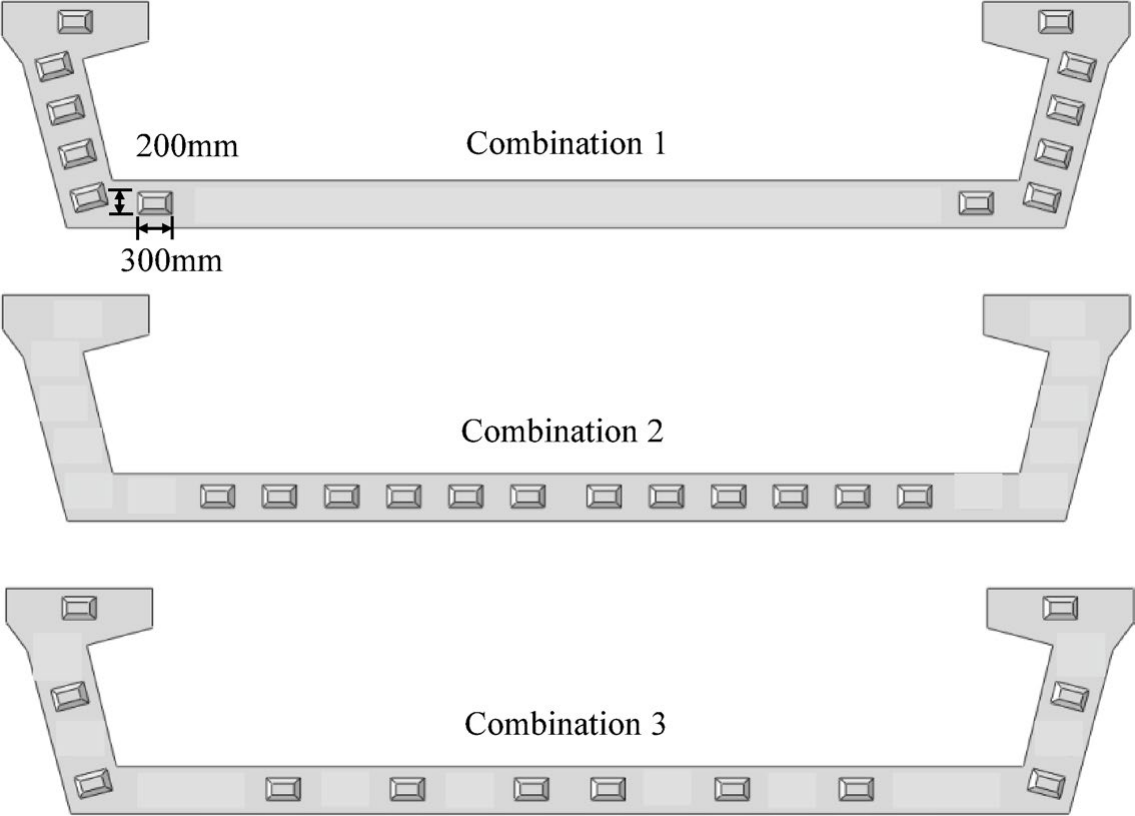
shear key arrangement scheme.

Three different finite element calculation models are established by changing the arrangement of shear keys. The parameters corresponding to models with different arrangement of shear keys are shown in Table 4. The arrangement of the shear keys used in the model, as shown in Fig. 11, each scheme has 12 shear keys. In Combination 1, all shear keys are on the web. In Combination 2, all are on the floor. In Combination 3, all shear keys are evenly distributed between the floor and web.

**Table 4:** Parameters corresponding to models with different arrangement of shear keys.

Name	*R*(m)	Number	l (mm)	w (mm)	h (mm)	α (°)
Combination1	200	12	300	200	50	45
Combination2	200	12	300	200	50	45
Combination3	200	12	300	200	50	45

**Fig. 12:**
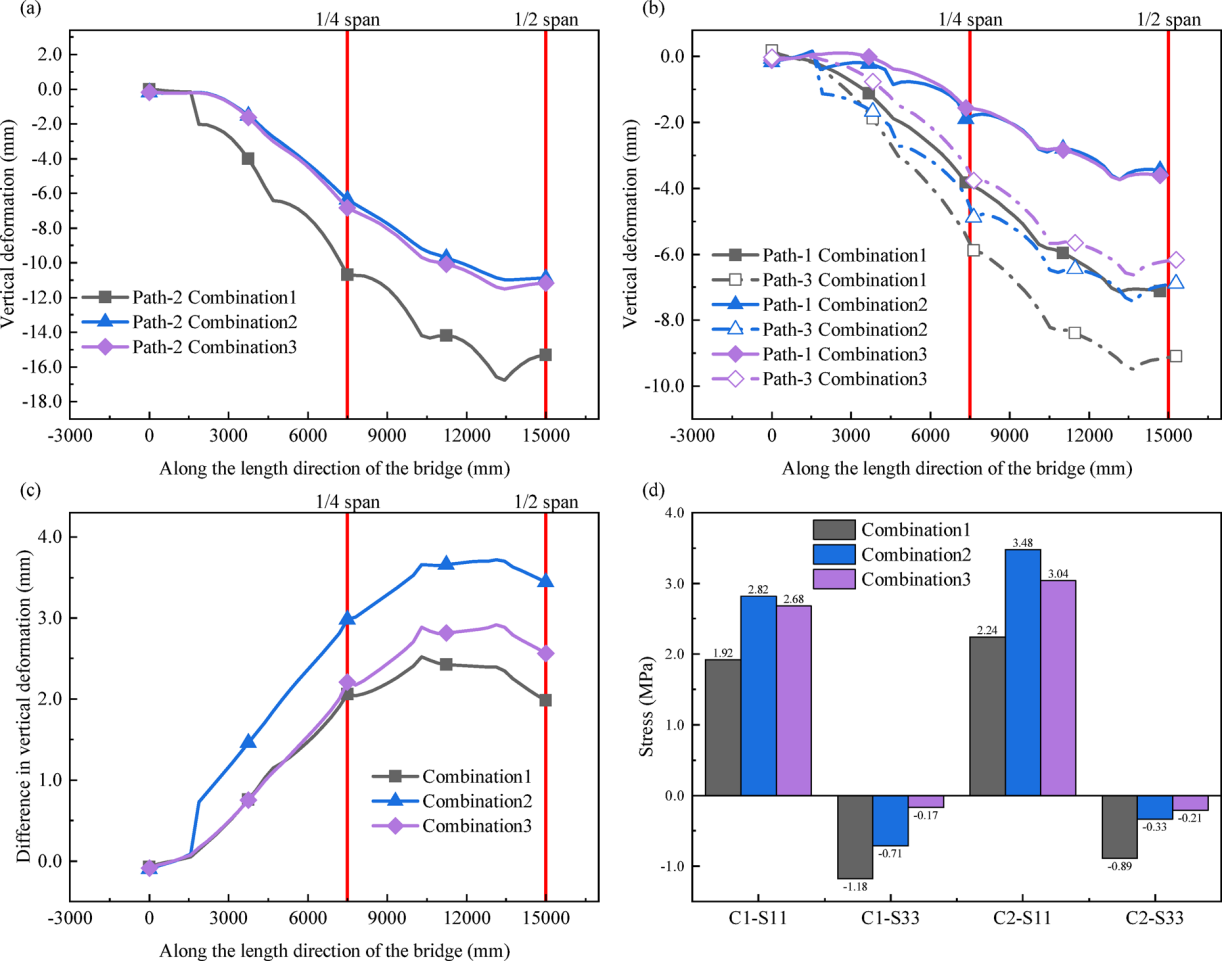
Effect of different shear key arrangements on bridge model calculation results. (**a**) Vertical deformation along Path-2; (**b**) Vertical deformation along Path-1 and Path-3; (**c**) The difference in vertical deformation along Path-1 and Path-3; (**d**) Stress at different bridge interfaces.

Combination1, Combination2 and Combination3 models are used for calculation and analysis, and discusses the effect of shear key arrangement on the torsional stiffness and interfacial stress of the bridge. The calculation result is shown in Fig. 12.

As shown in Fig. 12 (a) and (b), on Path-1 andPath-2, the vertical deformation of Combination 2 and 3 is basically the same and less than that of Combination 1. On Path-3, compared with Combination 1, the vertical deformation of Combination 2 and Combination 3 at the quarter span decreased by 17.05% and 35.86% respectively, and that at the mid span decreased by 24.24% and 32.20% respectively. Therefore, the arrangement of shear keys has a significant effect on the control of vertical deformation. Considering all three paths, Combination 3 is the most effective in controlling vertical deformation, followed by Combination 2, and Combination 1 has the worst effect.

As shown in Fig. 12 (c), there are significant differences in the vertical deformation difference between the inner and outer sides of the bridge under different shear key arrangement. Combination 2 has the largest vertical deformation difference, followed by Combination 3, and Combination 1 has the smallest vertical deformation difference. The arrangement of shear keys will affect the torsional deformation of bridges, with Combination 1 being the most effective in suppressing torsional deformation, followed by Combination 3, and Combination 2 being relatively ineffective. Therefore, setting more shear keys on the web is more beneficial to improve the torsional stiffness of the bridge.

Figure 12 (d) shows the bridge interfacial stress under three different shear key arrangements. For C1 and C2, the order of S11 from maximum to minimum is Combination 2, Combination 3, and Combination 1. The absolute values of S33 are in ascending order as Combination 3, Combination 2, and Combination 1. This shows that Combination 1 is the best in reducing the tangential stress, and has the maximum normal compressive stress. Therefore, the priority of setting shear keys on the web can enhance the integrity of the bridge and improve the shear bearing capacity of the joints.

In summary, increasing the shear key on the web can increase the torsional stiffness and improve the interfacial stress. The addition of shear keys on the floor is beneficial to increase the normal connection stiffness of the bridge interface, which should be comprehensively selected.

### The effect of shear key size

**Fig. 13:**
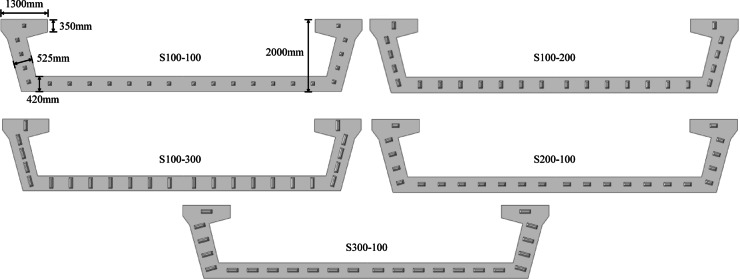
Layout of different size of shear keys.

Five different finite element calculation models are established by changing the size of shear keys. The parameters corresponding to models with different size of shear keys are shown in Table 5. The arrangement of shear keys is shown in Fig. 13. To avoid sharp corners in the shear keys, *h* has been changed from 50 mm to 30 mm^30^.

**Table 5:** Parameters corresponding to models with different size of shear keys.

Name	*R*(m)	Number	l (mm)	w (mm)	h (mm)	α (°)
S100-100	200	24	100	100	30	45
S200-100	200	24	200	100	30	45
S300-100	200	24	300	100	30	45
S100-200	200	24	100	200	30	45
S100-300	200	24	100	300	30	45

**Fig. 14:**
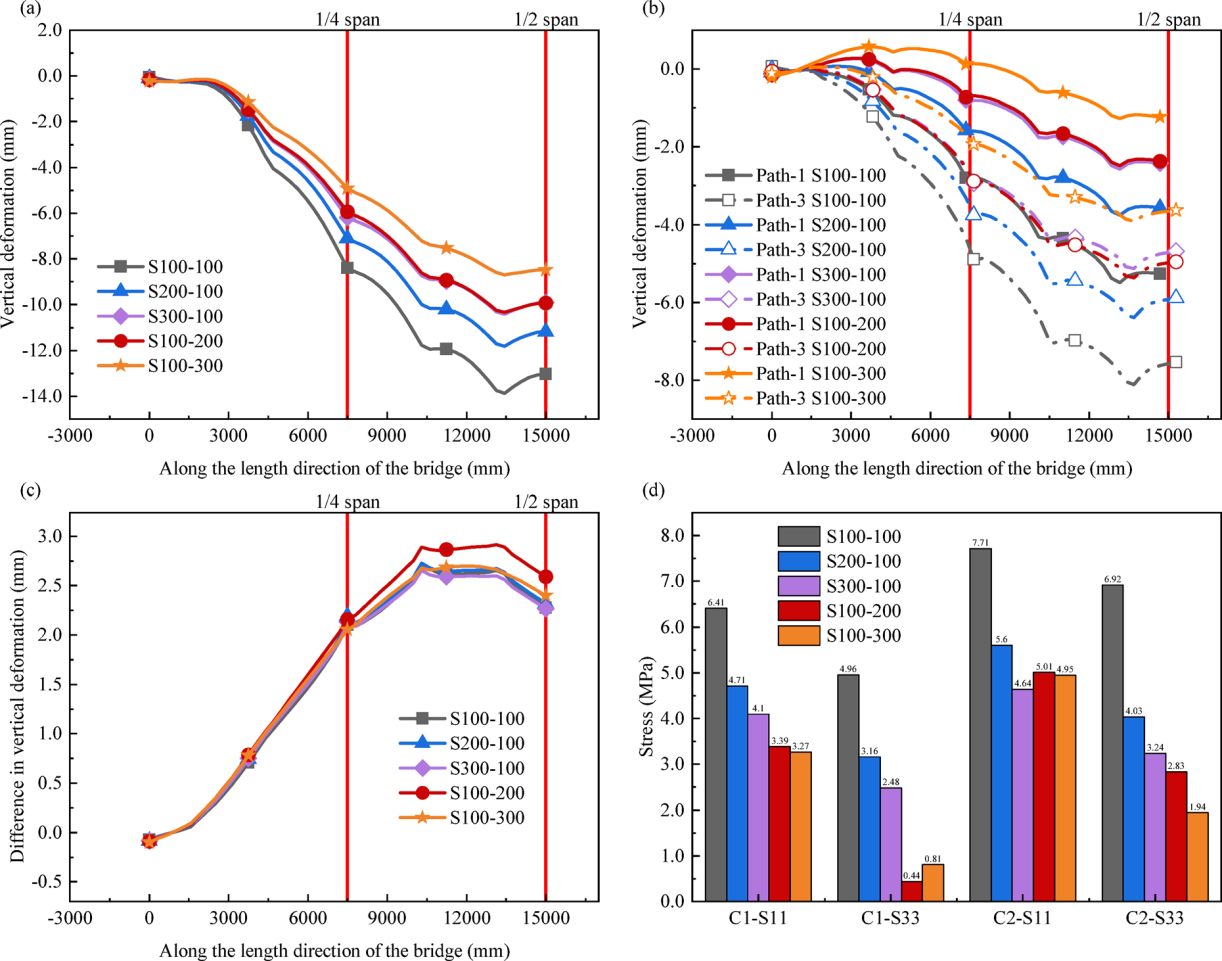
Effect of different shear key size on bridge model calculation results: (**a**) Vertical deformation along Path-2; (**b**) Vertical deformation along Path-1 and Path-3; (**c**) The difference in vertical deformation along Path-1 and Path-3; (**d**) Stress at different bridge interfaces.

The five models S100-100, S200-100, S300-100, S100-200 and S100-300 are used for the calculation and analysis, and the results are shown in Fig. 14.

As shown in Fig. 14 (a) and (b), as the size of the shear key gradually increases, the vertical deformation of the model along the three paths shows a decreasing trend. Taking path-2 as an example, when the width *w* of the shear key is kept constant at 100 mm, compared with the length *l* of 100 mm, the vertical deformation of the model at the quarter span is reduced by 15.40% and 26.43% respectively when *l* is 200 mm and 300 mm, and at the mid span the vertical deformation is reduced by 14.21% and 23.98% respectively. Similarly, when *l* remains constant, compared to *w* of 100 mm, the vertical deformation of the model at the quarter span is reduced by 29.46% and 41.43% at *w* of 200 mm and 300 mm, respectively, while at the mid span the vertical deformation is reduced by 23.76% and 34.95%, respectively. Although S200-100 and S100-200 have the same base area, the latter exhibits superior performance in controlling vertical deformation, with effects comparable to or even slightly better than S300-100. In addition, compared with S300-100, S100-300 exhibits more outstanding ability in controlling vertical deformation. Therefore, increasing the size of shear keys can effectively reduce the vertical deformation of bridges. Under the same foundation area, increasing the width of shear keys is more effective in controlling vertical deformation than increasing their length.

According to the key conclusions of Hertzian contact theory, for two elastic bodies under normal force, the maximum contact stress is positively correlated with the normal force and negatively correlated with the contact area. Therefore, when the width of the shear key is increased, the contact area of the shear key in the direction of vertical displacement expands directly. A larger contact area in this direction enables more efficient transmission of vertical shear force, thereby reducing relative slip between segments. In contrast, increasing the length of the shear key expands the lateral contact area, which is not the main direction of displacement when the segments are stressed, thus making a weaker contribution to suppressing vertical deformation^31–33^.

Figure 14 (c) indicates that the variation in shear key size has little effect on the difference in vertical deformation between the inner and outer sides of the bridge. However, increasing the size of the shear keys slightly helps to improve the torsional stiffness of the bridge by carefully comparing the subtle differences in the curves. Under the same basic area, longer shear keys provide slightly stronger torsional stiffness.

Observing Fig. 14 (d), different shear key size configurations will significantly affect the stress at the bridge interface. When *w* is constant and *l* increases from 100 mm to 200 mm and then to 300 mm, both S11 and S33 at C1 and C2 decrease. Similarly, when *l* is fixed and *w* increases from 100 mm to 200 mm, the stresses reduce significantly. However, increasing *w* from 200 mm to 300 mm results in a smaller decrease in S11 and even a slight increase in S33 at C1, while S33 at C2 continues to decrease significantly. This also confirms that the sensitivity of different interfaces of bridges to changes in shear keys varies. Under the same substrate area, widening the shear key can more effectively reduce interfacial stress than lengthening the shear key. This indicates that when reducing the stress at the bridge interface, increasing the width of the shear keys can be prioritized to achieve lower stress levels.

Increasing the size of the shear key can effectively reduce the vertical deformation and interfacial stress of the bridge. Under the same base area, increasing the width of the shear key can more effectively control the vertical deformation and reduce the interfacial stress than increasing length. Although longer shear keys provide slightly stronger torsional stiffness under the same base area, overall, the contribution of increased shear key size to improving bridge torsional stiffness is still not significant.

## Conclusions

This study systematically analyzes the effects of curvature and shear key design of segmental U-shaped curved bridge on torsional stiffness and interfacial stress of the bridge. The main conclusions are as follows:

1. Regarding improving torsional stiffness: The radius of curvature has a significant impact on torsional stiffness. As the radius increases, the torsional deformation of the bridge will gradually decrease. For curved bridges with a radius ranging from 200 to 400 m, it is recommended to adopt a radius of no less than 300 m; for bridges with a radius of no more than 200 m, since placing shear keys on the web can maximize torsional stiffness, 80% or more of the shear keys can be arranged on the web, and the rest can be evenly distributed on the floor to compensate for torsional stiffness.
2. Regarding improving interfacial stress: Increasing the number of shear keys and enlarging their size helps improve the interfacial stress state and enhance connection stiffness; and under the condition of the same base area, increasing the width is more effective than increasing the length. Therefore, it is recommended to prioritize increasing the width of the shear keys, which should not be less than 1/2 of the floor height, while increasing the number of shear keys as much as possible. For the bridge section studied in this paper, the number of shear keys are recommended to have no less than 16.

## Data Availability

The authors confirm that the data supporting the findings of this study are available within the article.
